# Automated Travel History Extraction From Clinical Notes for Informing the Detection of Emergent Infectious Disease Events: Algorithm Development and Validation

**DOI:** 10.2196/26719

**Published:** 2021-03-24

**Authors:** Kelly S Peterson, Julia Lewis, Olga V Patterson, Alec B Chapman, Daniel W Denhalter, Patricia A Lye, Vanessa W Stevens, Shantini D Gamage, Gary A Roselle, Katherine S Wallace, Makoto Jones

**Affiliations:** 1 VA Salt Lake City Health Care System US Department of Veterans Affairs Salt Lake City, UT United States; 2 Division of Epidemiology Department of Internal Medicine University of Utah Salt Lake City, UT United States; 3 Department of Rocky Mountain Cancer Data Systems University of Utah Salt Lake City, UT United States; 4 National Infectious Diseases Service Specialty Care Services US Department of Veterans Affairs Cincinnati, OH United States; 5 Division of Infectious Diseases Department of Internal Medicine University of Cincinnati College of Medicine Cincinnati, OH United States; 6 Cincinnati VA Medical Center US Department of Veterans Affairs Cincinnati, OH United States; 7 Office of Biosurveillance Veterans Affairs Central Office US Department of Veterans Affairs Washington, DC United States; 8 National Biosurveillance Integration Center Countering Weapons of Mass Destruction Department of Homeland Security Washington, DC United States

**Keywords:** natural language processing, machine learning, travel history, COVID-19, Zika, infectious disease surveillance, surveillance applications, biosurveillance, electronic health record

## Abstract

**Background:**

Patient travel history can be crucial in evaluating evolving infectious disease events. Such information can be challenging to acquire in electronic health records, as it is often available only in unstructured text.

**Objective:**

This study aims to assess the feasibility of annotating and automatically extracting travel history mentions from unstructured clinical documents in the Department of Veterans Affairs across disparate health care facilities and among millions of patients. Information about travel exposure augments existing surveillance applications for increased preparedness in responding quickly to public health threats.

**Methods:**

Clinical documents related to arboviral disease were annotated following selection using a semiautomated bootstrapping process. Using annotated instances as training data, models were developed to extract from unstructured clinical text any mention of affirmed travel locations outside of the continental United States. Automated text processing models were evaluated, involving machine learning and neural language models for extraction accuracy.

**Results:**

Among 4584 annotated instances, 2659 (58%) contained an affirmed mention of travel history, while 347 (7.6%) were negated. Interannotator agreement resulted in a document-level Cohen kappa of 0.776. Automated text processing accuracy (F1 85.6, 95% CI 82.5-87.9) and computational burden were acceptable such that the system can provide a rapid screen for public health events.

**Conclusions:**

Automated extraction of patient travel history from clinical documents is feasible for enhanced passive surveillance public health systems. Without such a system, it would usually be necessary to manually review charts to identify recent travel or lack of travel, use an electronic health record that enforces travel history documentation, or ignore this potential source of information altogether. The development of this tool was initially motivated by emergent arboviral diseases. More recently, this system was used in the early phases of response to COVID-19 in the United States, although its utility was limited to a relatively brief window due to the rapid domestic spread of the virus. Such systems may aid future efforts to prevent and contain the spread of infectious diseases.

## Introduction

Epidemiologic clues are critical to understand how infectious diseases spread. When working up a rapidly unfolding event, a history of travel to an endemic region can be a valuable piece of evidence for public health authorities and biosurveillance experts who must often work quickly in tracing linkages to manage outbreaks. When information about travel to endemic areas is present, it may confirm an existing understanding or flag an epidemiologist to gather additional information. Alternatively, an absence of travel records for patients who are infected may suggest the possibility of local transmission. Even on an individual patient level, the importance of evaluating travel history has been previously recognized as crucial, in addition to symptoms, in establishing an appropriate differential diagnosis and optimizing testing [1,2].

Unfortunately, information about patient travel in the electronic health record (EHR) is still not typically recorded in a structured format but in unstructured clinical documents [3], especially when screening questions have not been mandated for emergency department triage. Instead, much of this information can be recorded in notes by specialists or others who suspect a travel-related disease. Although important, the need for significant annotated data and an effective document selection process are likely reasons why automatic extraction of travel history from clinical documents has not been explored extensively [4]. It has been noted that no standard tools exist for identifying critical information. Several gaps remain in gathering crucial data including accurate and timely patient travel history [5]. Reliable methods are needed for automated identification of such information from documents.

Machine learning has been applied increasingly to information extraction tasks from text. Recent developments allow scaling to large amounts of data while achieving accurate results. Such methods have proven useful to identify hate speech in social media [6] and for accelerating information gathering from legal documents [7]. These advancements have shown to be effective in several aspects of health care ranging from automated processing of radiology reports [8] to detection and relation of adverse drug events [9].

Previous work has identified geographic locations in text such as newswire articles and social media posts [10,11]. Biomedical literature has been previously used to extract geolocation information for infected hosts to enable virus spread modeling [12]. Although other means of identifying location exist, such as mobile apps, challenges remain to use these resources in health care, including privacy concerns and integration with clinical indicators of disease such as laboratory results recorded in the EHR. Using EHR documentation to identify patient travel is rarely reported potentially due to the complexity of the task.

In health care, a comprehensive understanding of locations visited by a patient is important for understanding key epidemiologic links. This information is especially crucial for respiratory diseases, which can be spread rapidly through international travel [1]. Most notably, early efforts in managing the spread of COVID-19 have relied upon information about travel to prevent the spread of the virus [13-15]. Several recent studies have leveraged publicly available data resources such as social media to conduct surveillance of the virus including travel history, symptoms, and concerns [16-18].

In this study, we created a reference standard for detection of patient travel history from the EHR through manual chart abstraction, developed an automatic text extraction pipeline, and deployed the system in preparedness for public health threats. The goal of the system in 2017 and 2018 was not only to evaluate the travel in the context of Zika and other emerging arboviral diseases at the time but also with the intent to expand the system to support the surveillance of other emerging infectious diseases. To that effect, this capability was used during a brief window to monitor the initial spread of COVID-19 infections in the United States.

## Methods

### Data Set

The Veterans Affair (VA) Corporate Data Warehouse contains clinical data from 170 medical centers and over 1000 outpatient sites across the United States [19]. This study was developed with Corporate Data Warehouse data from 2015 to 2017 across more than 6.4 million patients with over 694 million clinical notes. This study was conducted in VA to provide tools to facilitate biosurveillance and health care operations. After the initial development, accuracy was evaluated on a sample of 57 patients whose documents were authored up to April 2018. This system currently continues to run for operational biosurveillance data insights.

### Annotation Guideline

The annotation guideline was developed in collaboration with epidemiologists and natural language processing (NLP) researchers. The scope of annotation was to collect past travel history and differentiate it from future or hypothetical travel discussion. Any reason for travel was allowed, including military deployment or medical tourism. No restrictions were made on the level of geographic specificity. Mentions of international travel vary in the level of geographic specificity. Although some travel locations are mentioned precisely (eg, “Mexico City”), others include landmarks (eg, “Great Barrier Reef”) or free-form description (eg, “beach in the Mexican Riviera”). This annotation scope was simplified into one sentence to enable rapid annotation: “Does this text indicate that the patient has been somewhere outside the United States?”

### Annotation Corpus Selection

**Figure 1:**
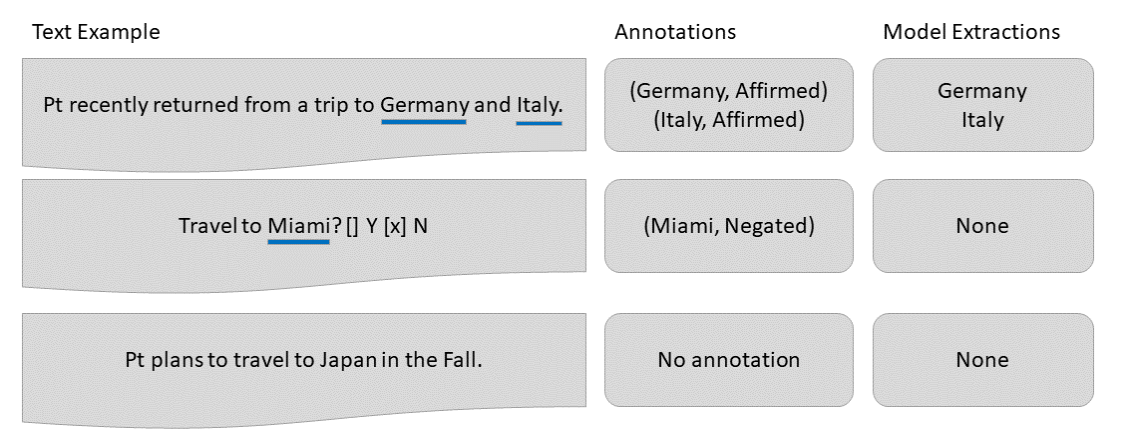
Illustration of this study's contributions. Included here are synthetic text examples, annotation output format, and model extraction of positive location mentions now deployed for public health response.

To train an automated text pipeline, we annotated a corpus for travel history mentions. Some examples are illustrated in Figure 1. Since clinician documentation of patient travel history in the EHR may vary, we used a strategy to identify likely candidate statements. The cohort for these potential travel mentions were all patients who received laboratory testing for the arboviral diseases Zika, dengue, or chikungunya from January 2015 to April 2017, a date range that corresponded with concerns about Zika virus transmission. For this cohort, we included clinical notes authored between 1 month before and 6 months after lab specimens were taken.

To further reduce the annotation corpus for relatively rare events, a semiautomated process was implemented similar to previous work [20]. The first step involved seed terms and phrases likely related to travel. These terms were comprised of cities and countries from the GeoNames gazetteer [21]. This also included phrases likely related to travel history, such as “recently returned from X” or “traveled on a cruise to X.” This set also included curated patterns for travel history questions common in the EHR. Some questions asked patients about relatively recent concerns in 2017 such as Zika or dengue. Meanwhile, others included questions about past travel related to Ebola or Middle East respiratory syndrome. These question patterns were not used as inclusion criteria for the annotation corpus since this remained limited to arboviral testing patients.

**Figure 2:**
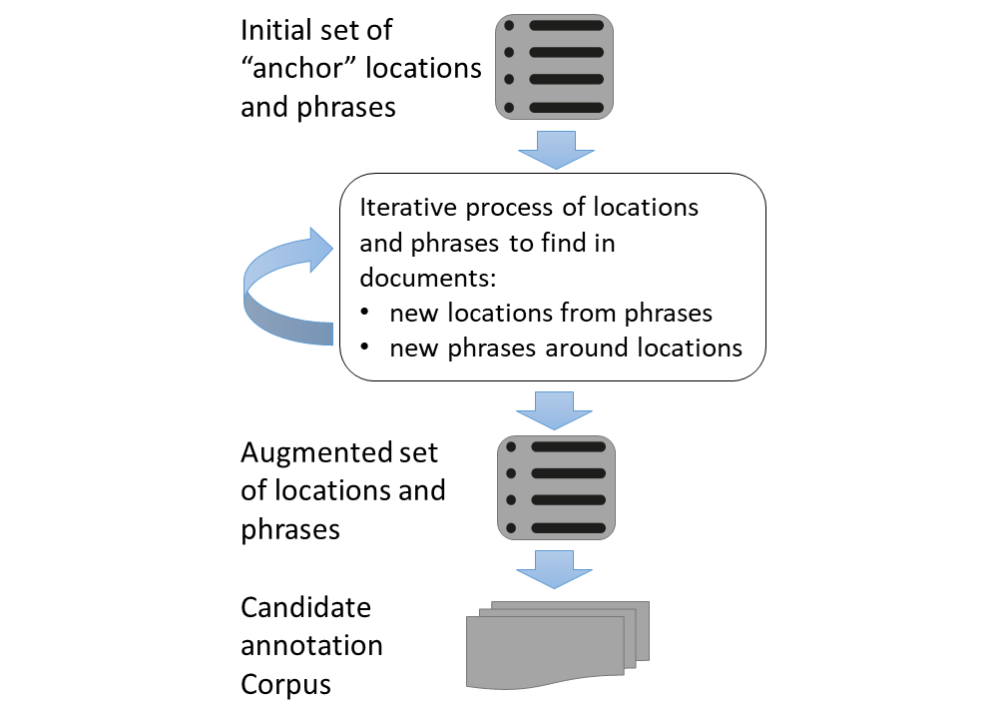
Bootstrapping process to identify previously unknown locations and phrases to enrich a document set for travel history mentions.

Next, these initial terms and phrases were used to retrieve documents from the patient cohort, which received arbovirus lab testing. An iterative bootstrapping approach was then used to collect additional documents. This approach was chosen to efficiently identify patterns from a large corpus of notes. At each iteration, new travel location terms and travel-related phrases expanded the initial seed terms, as these were extracted in a semiautomated fashion illustrated in Figure 2.

New travel locations were found when co-occurring with detected travel phrases. Likewise, travel location matches were used to extract new travel-related phrases by inspecting n-grams collocated with locations such as “10 day vacation to X...” and “symptoms after a 12 hour flight from X...” Where possible, these phrases were generalized to regular expressions. Locations of varying specificity and lexical variation were gathered, such as “the big island,” “porto rica,” and “deep Mexico.” This process was repeated several times until a set of candidate documents had been gathered for annotation.

For rapid annotation, short snippets of three sentences were collected where one of the sentences contained a location, phrase, or question template. Document retrieval and sentence retrieval rules were implemented using the Leo framework [22]. These snippets were then deduplicated by performing exact lexical comparison with no preprocessing.

### Annotation Methods

Annotation was performed by three annotators and consisted of span level annotation on short snippets in ChartReview [23]. In each reviewed snippet, annotators highlighted the span of a travel location mention and specified whether the location was affirmed to be a location that the patient visited or if travel was negated (eg, the patient did not visit the location or denied such visits). Examples of text and annotation data are presented in Figure 1.

Annotation agreement was measured on a set of 100 snippets annotated by two annotators. Agreement was measured at a span level of locations and at a record level of travel history in a snippet.

Location agreement was calculated for all annotated location text spans and required an exact match of text offset and negation status. Any difference in status was counted as a disagreement and any difference in text span was considered as a separate annotation element. Record agreement combined any annotated location status so that each snippet would be assigned a class of either no travel mentioned, negated locations, positive locations, or mixed.

### Extraction Methods

A text processing pipeline was built to test feasibility of travel history extraction for operational use. This pipeline used models trained from the reference standard created from arboviral illness cases. The objective of this pipeline is to label affirmed travel location mentions. Although negated locations were gathered in annotation, these were not used in model training. Figure 1 provides an example of this consideration.

Given a requirement for rapid processing of documents with available computing resources, the model was constructed using conditional random fields (CRF) implemented in CRFSuite in Python [24,25]. Classes of contextual features around tokens were evaluated.

**Figure 3:**
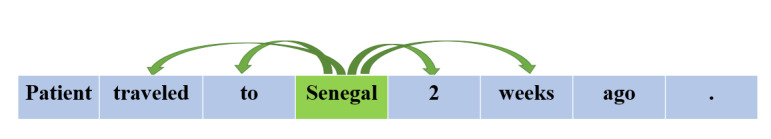
Illustration of feature encoding window for the token "Senegal" in an example sentence.

Annotated documents were split into 80% training, 10% validation, and 10% test to be held out from any training or evaluation until the final experiments. Feature types and model hyperparameters (eg, token window size) were evaluated via random search and cross validation using scikit-learn [26]. The set of features in this model include tokens, lemmas, character n-grams, part of speech tags, token shape (ie, capitalization, digits, and punctuation), gazetteer match of cities and countries from GeoNames [21], and word embedding clusters [27,28]. An example illustrating feature encoding is presented in Figure 3 and Table 1. Part of speech tags (eg, VBD) are defined as per the Penn Treebank tag values [29].

**Table 1:** Feature encoding examples for the token “Senegal” in Figure 3.

Feature class	Encoding examples^a^
Tokens	-2_token: ‘traveled’; -1_token: ‘to’; 0_token: ‘senegal’; 1_token: ‘2’; 2_token: ‘weeks’
Lemmas	-2_lemma: ‘travel’; -1_ lemma: ‘to’; 0_ lemma: ‘seneg’; 1_ lemma: ‘2’; 2_ lemma: ‘week’
Token shape	-2_islower: 1; -1_islower: 1; 0_iscapital: 1; 1_isdigit: 1 2_islower: 1
Part of speech [29]	-2_pos: ‘VBD’; 1_pos: ‘TO’; 0_pos: ‘JJ’; 1_pos: ‘CD’; 2_pos: ‘NNS’; -2_-1_pos: ‘VBD_TO’; -1_0_pos: ‘TO_JJ’; […]
Character n-grams	[...]; 0_char_bigram_se: 1; 0_char_bigram_en: 1; [...]; 0_char_trigram_sen: 1; 0_char_trigram_ene: 1; [...]
Gazetteer match	-2_gaz: 0; -1_gaz: 0; 0_gaz: 1; 1_gaz: 0; 2_gaz: 0
Word embedding clusters	-2_cluster: ‘6101’; -1_cluster: ‘8804’; 0_cluster: ‘5470’; [...]; -2_-1_cluster: ‘6101_8804’; -1_0_cluster: ‘8804_5470’; [...]

^a^Single quoted values are encoded as categorical features rather than ordinal. When present, a prefix ‘-2_’ indicates features for the token 2 to the left, ‘0_’ for the current token, etc.

Model performance was evaluated with classification metrics of precision, recall, and F1 as well as bootstrapped sampling methods at a 95% CI [30,31]. All reported findings here used strict matching such that if the annotator marked a multi-word visited location such as “Western Africa,” both tokens must be detected.

As a comparison, we selected two general purpose location named-entity recognition (NER) models, which were evaluated for geoparsing. Since these systems were developed for geolocation detection and not travel history, the intent of this comparison is to provide motivation that the task of travel history is distinct from geoparsing. In one comparison experiment, we applied the NER model from the Stanford NLP library to the annotated travel corpus [10]. In the other experiment, we used spaCy [32] to label the location entity. Although these are general-purpose models for location extraction, they have been used in several system comparisons for parsing geographic locations from text [33]. Among the potential libraries reviewed in [33], these were selected given convenient usage in the Python programming language. As this was a simple experiment to provide motivation of this being a distinct task, these systems were not retrained or augmented with gazetteer entries, as they were applied to the annotated data set with no modifications. Spans labeled as locations by each baseline model were used in this simplified evaluation of identifying patient travel location. Two comparisons were made since these existing systems do not allow for an ideal comparison. One was limited to locations annotated for past affirmed travel and the other for any annotated location, which would include negated, future, or hypothetical travel.

### Postdeployment Evaluation Methods

After an initial model had been deployed for operational purposes, an analyst with clinical experience performed a chart review to estimate system performance. This evaluation was performed to account for a potential selection bias due to the keyword-based selection of records for the original reference standard and to evaluate the system on more recent data not available during the training phase.

To conduct this evaluation, 57 unique patients were randomly selected among patients who received laboratory testing for Zika, dengue, or chikungunya prior to April 2018. For each patient, clinical documents were reviewed surrounding the dates of the specimen acquisition and test completion. This review resulted in the manual gathering of past affirmed patient locations. These locations were compared against automated locations identified in operational reporting.

### Ethics Approval

This analysis was performed under project approval from the University of Utah Institutional Review Board and the VA Salt Lake City Health Care System Research and Development Office.

## Results

### Annotation Corpus Selection Results

The process for selecting candidate travel history mentions was initiated on 250,133 clinical documents among 2274 unique patients who received arbovirus testing. The mean age of these patients was 59 years, and 1960 (86.2%) patients were male. A total of 6482 snippets were identified as potential travel mentions, and 3894 (60.1%) were acquired from locations, 2576 (39.7%) from phrases, and 12 (0.2%) from template questions.

After removing duplicates, 4584 unique snippets remained to be annotated. Since there were over 1800 duplicates in the candidates, some of the most frequent are explained here. Many of the duplicate snippets consisted of several travel question templates, which were identical in hundreds of snippets across multiple patients, such as “Has the patient traveled to an Ebola affected area? [] Y [X] N” or “Has the patient traveled to West Africa?” Other duplicates included verbatim travel history records that had been pasted into the same patient’s medical record multiple times across separate documents. Other frequent duplicate snippets matched location strings yet were involved in unrelated common clinical templates such as “Sleep Disorders: Berlin and Epworth Questionnaires.”

### Annotation Results

Of the 4584 unique snippets annotated, 2659 (58.0%) were annotated as affirmed travel history and 347 (7.6%) were marked as negated. The remaining 1578 (34.4%) snippets were annotated as not containing any mention of travel history.

Both location and record agreement were calculated using Cohen kappa [34]. In the set of 100 double annotated snippets, span agreement was measured as κ=0.706 and record agreement as κ=0.776.

Documentation of patient travel history in these annotations vary in a spectrum from semistructured questionnaires (eg, “Have you visited a region known for Zika transmission?” “Has the patient recently returned from Brazil, Mexico or Miami, Florida? [] Y [X] N”) to coarse grain descriptions (eg, “went to Europe”) to detailed traces of travel activity (eg, “returned to the United States on July 2 from Guatemala by way of Mexico City (July 1)”).

Instances of annotator disagreement were examined to identify the categories of differences and explore potential refinement for the annotation guideline. Several disagreements stemmed from differing attribution of past affirmed travel as opposed to future or hypothetical travel. For example, in one synthetic example, one annotator marked the mention “...travelling to visit sister in Hungary in May” as future travel, while another marked this as past affirmed travel. These errors were the most frequent cause for disagreement.

Additional challenges in this task occurred as this study was conducted using Department of VA records, where military deployment is common but actual patient exposure may remain ambiguous. For example, a template such as “Service Era: Vietnam” does not necessarily imply that the patient was in Vietnam. Statements like these on their own did not lead to much disagreement, yet we observed that annotators made different inferences in more complicated examples. In short snippets, longer distance statements could lead to differing interpretations. For example, in a snippet such as “Service: Persian Gulf War...<another long sentence>...but after coming home and getting married...,” one annotator inferred that the patient had been in the Persian Gulf region while the other did not.

Geographic specificity also led to some annotation disagreement. Although the annotation gave no requirement for specificity, there were instances where a sentence like “Pt traveled outside the United States from early May to August” was marked by one annotator as a past affirmed travel location “outside the United States.” Although the value of such travel history could be debated for the purposes of infectious disease surveillance, additional examples in the annotation guideline could have provided clarity in these situations.

The annotation identified 561 distinct locations across 8127 location spans. The most frequently annotated affirmed and negated locations are listed in Table 2. These locations exhibit a range of geographic specificity. Some mentions are specific cities or countries, while others are entire continents or regions (eg, Africa, West Africa) and others are highly specific such as the name of a beach resort or an oceanic cruise itinerary. Some location mentions are ambiguous, such as “Jamaica,” which could refer to the country or the neighborhood in New York City.

**Table 2:** Most frequently affirmed and negated annotated locations and their percentage of the 8127 total location text spans annotated.

Locations		Annotations, n (%)
**Affirmed**		
	Iraq	471 (5.8)
	Mexico	374 (4.6)
	Vietnam	251 (3.1)
	Costa Rica	251 (3.1)
	Dominican Republic	244 (3.0)
	Afghanistan	236 (2.9)
	Jamaica	187 (2.3)
	Puerto Rico	179 (2.2)
**Negated**		
	Liberia	341 (4.2)
	Guinea	341 (4.2)
	Sierra Leone	333 (4.1)
	Democratic Republic of Congo	130 (1.6)
	West Africa	114 (1.4)
	Mali	114 (1.4)
	Nigeria	89 (1.1)
	Western Africa	81 (1.0)

### Extraction Results

The best performing model to label affirmed travel history mentions was evaluated on the test set as 88.0% precision, 83.3% recall, and 85.6% F1 measure. A comparison to two existing systems in detecting past affirmed travel is presented in Table 3. Another example of these systems for any annotated travel location mentions, whether affirmed, past, negated, or future, is provided in Table 4. Although neither comparison is ideal, these differences highlight that identifying travel history is distinct from typical geolocation recognition.

**Table 3:** Comparison of our model to two other general-purpose baselines in the task of identifying past affirmed travel locations.

Model	Precision	Recall	F1 score
Stanford location NER^a^	40.2	76.7	52.8
spaCy NER	36.0	62.0	45.5
Proposed model	88.0	83.3	85.6

^a^NER: named-entity recognition.

**Table 4:** Comparison of the two other general-purpose baselines in the task of identifying any annotated travel location, whether affirmed, negated, or future.

Model	Precision	Recall	F1 score
Stanford Location NER^a^	62.3	81.7	70.7
spaCy NER	52.5	63.4	57.5

^a^NER: named-entity recognition.

Feature importance of our proposed model was measured as an ablation experiment quantifying contribution of each feature set with respect to a baseline set of surface-level features. The results of this feature ablation are presented in Table 5.

**Table 5:** Ablation results of model features.

Feature set	Precision (95% CI)	Recall (95% CI)	F1 score (95% CI)
Tokens, lemmas, token shape	85.0 (80.8-87.0)	69.1 (64.5-74.1)	76.3 (72.7-79.5)
+ Part of speech	87.4 (83.3-90.5)	72.2 (67.6-76.9)	79.1 (75.8-82.1)
+ Character n-grams	85.9 (82.1-89.4)	77.2 (72.9-81.1)	81.3 (78.0-84.3)
+ Gazetteer match	84.1 (79.9-87.1)	75.2 (70.9-79.7)	79.4 (76.0-82.6)
+ Word embedding clusters	87.6 (83.7-90.9)	74.9 (70.7-79.3)	80.8 (77.3-83.7)
All features	88.0 (84.2-90.9)	83.3 (78.9-86.6)	85.6 (82.5-87.9)

Since hyperparameter values such as token window size and the size of character n-grams have an impact on model performance, some of these were varied to identify the optimal parameters and quantify sensitivity to value changes. These values did not appear to cause much variance in precision but did improve recall. The best performing model integrated features within a window size of 2 tokens and character n-grams of size 2 and 3. This analysis is presented in Table 6.

**Table 6:** Results of varying certain feature extraction hyperparameters.

Parameter difference	Precision (95% CI)	Recall (95% CI)	F1 score (95% CI)
Window size=1	87.8 (84.3-91.2)	80.5 (76.7-84.3)	84.1 (80.9-86.4)
Window size=3	87.4 (83.9-90.9)	79.2 (74.8-83.6)	83.1 (79.7-86.1)
Character n-gram size=2	87.9 (84.5-91.1)	82.7 (79.0-86.4)	85.2 (82.6-88.1)
Character n-gram size=3	87.9 (84.0-91.0)	81.2 (77.4-84.8)	84.4 (81.4-87.0)
Character n-gram size=4	87.9 (84.1-91.0)	81.0 (77.1-84.6)	84.3 (81.6-86.9)
Optimal hyperparameters (window size=2, character n-gram sizes=2 and 3)	88.0 (84.2-90.9)	83.3 (78.9-86.6)	85.6 (82.5-87.9)

Since this pipeline has been deployed for running daily operations, we have measured the total processing time for querying source documents, processing them, and storing the results back to the database. In February 2020, responding to the spread of COVID-19, over 978,000 documents were processed with a median length of 2414 characters. Throughput for the system is an average of 2.01 documents per second using a single machine core (ie, Intel Xeon E5-4650 @ 2.10 GHz).

### Postdeployment Evaluation Results

Evaluation of the system on a previously unseen sample of patients and documents following deployment resulted in an estimated system performance of 71.9% precision, 78.1% recall, and 74.9% F1 measure. In consultation with infectious disease analysts, the measured performance was determined to be not sufficient, so the newly created annotations were used to perform an error analysis of model predictions. Several of the false positives were instances in which the initial model predicted future or negated travel. Many of the false negatives were locations whose spelling did not match canonical gazetteer entries. Besides these errors, the annotated corpus was reviewed, and some annotations were adjudicated and modified. Several of these adjudicated annotations occurred in statements of future or hypothetical travel, indicating that the guideline may benefit from refinement. Additional feature types were evaluated including word embedding clusters and character n-grams. Ablation experiments were performed to determine the best feature set. Before these improvements, the model’s performance on the test split of annotated documents was 82.9% precision, 76.4% recall, and 79.5% F1 measure. The improved model exhibited a 6.1% increase in F1 and is reported in the preceding section.

## Discussion

### Principal Findings

In this study, we have explored and demonstrated the feasibility of extracting patient travel history from clinical documentation of a large national cohort. Relying on semiautomated methods for lexicon expansion, we manually labeled a data set to train an extraction model. Our findings demonstrate that training an accurate model to extract travel mentions is feasible in an automated system. Both labeled sets and the modeling approaches were chosen to minimize development time and computational resources necessary to continue surveillance in day-to-day operations. The baseline comparison presented here is a simplified evaluation, but it demonstrates that general-purpose geoparsing solutions alone result in lower precision. This is due to these systems labeling all locations in text, while the task in this study was to identify past affirmed travel for public health response. Our system has been deployed for operational use.

Since graphics processing unit acceleration is not currently available in the computing environments where system development was conducted and models later deployed, more costly model options such as recurrent neural networks and contextual language models (ie, Bidirectional Encoder Representations from Transformers, Generative Pre-trained Transformer 2, and Universal Language Model Fine-tuning) have not yet been evaluated [35-37]. Rather, we present a linear model that leverages information from neural language models that can be rapidly trained on widely available hardware. Our system has been deployed on a central processing unit to support health care operations churning through large volumes of clinical documents daily. Since deployment in November 2017 through July 2020, more than 18 million documents have been processed as part of a daily data pipeline.

Our proposed system exhibits promising performance of location mention retrievals in text. Future work may attempt to disambiguate location text or resolve mentions to a geolocation. Additionally, annotation of timing or duration of travel could provide another layer of travel history. We have not yet measured how frequently such information is recorded in EHR, but we hope to answer this question in a future study.

Further, although negated location mentions were annotated for the reference standard, these were not used at the time of training. The extraction model may be improved by distinguishing between affirmed and negated locations, and public health efforts could be assisted by such information when conducting efforts to rule out exposures.

Originally deployed in November 2017, this system has been used in VA to rapidly respond to public health events. Given that this system processes millions of notes each week, it can be integrated with other biosurveillance metrics to give a sense to decision makers about the extent that travel history may be playing a role in disease dynamics. These higher-level metrics include the absolute counts and percentages of emergency department visits with respect to syndromic categories such as respiratory illness, gastrointestinal conditions, and diseases.

After this system was deployed, it was initially used by VA biosurveillance analysts to rapidly screen new cases of Zika in the nation. Rapid visibility of automated travel history extractions allowed analysts to scan new cases and perform additional chart review daily if there was no travel history in the patient profile. For example, if a new case of Zika occurred for a patient living in a midwestern state without an automated travel profile, the next step could be to perform a chart review to determine whether the case might be reflective of local transmission. Since chart review can be a time-consuming process, the fact that many patients already had a travel profile to an endemic area meant that less time was consumed in manual review. Cases that did have a profile could be easily verified by performing keyword searches stemming from locations visited.

This system was already running in early 2020 such that it could be leveraged in response to the spread of COVID-19 in the United States. This capability was useful in early stages of transmission, as it was able to identify mentions of travel to endemic areas.

One way that this extraction was useful was by setting up broad surveillance and then using travel as additional criteria. Specifically, case review could be prioritized using case definitions for respiratory and influenza-like illness we had developed for broad syndromic surveillance. These definitions required both relevant International Classification of Diseases, Tenth Revision (ICD-10) diagnostic coding and concepts for signs and symptoms extracted from chief complaint text as concept unique identifiers (CUI) in the Unified Medical Language System [38]. ICD-10 codes were drawn from resources provided by the International Society for Disease Surveillance [39]. CUI values for these broad syndromic definitions were collected from prior work [40]. Combining multiple aspects of clinical data with travel information permitted rapid selection of cases to review in January 2020. During February 2020, as testing for COVID-19 became more standard, combining orders or results for such testing and travel regions permitted more specific case review.

This study was limited as the selection of annotation corpus was performed by location keywords and travel phrases. This could entail a bias in the location types and variants used in annotation and model training. We attempted to address these limitations of the annotation corpus by performing manual chart review and random sampling. This review was limited by resources, so future work may ensure that the annotation corpus is free of potential bias by performing further review by random sampling or other strategies leveraging clinical evidence. Although acceptable, the relatively low level of annotator agreement may be a potential limitation of our system. Additionally, preliminary data from corpus selection indicates that past affirmed travel mentions in clinical documents are likely to be imbalanced. Given this, experiments in ongoing work may achieve better model performance by exploring class imbalance strategies such as undersampling or oversampling.

The utility of this system for COVID-19 specifically was limited to a relatively brief window in the early phases of transmission when containment was possible and travel was a pertinent risk factor. The Centers for Disease Control and Prevention (CDC) guidance for Persons Under Investigation on February 12, 2020, included explicit mention for travel to Wuhan or Hubei Province [41]. By March 4, the CDC removed these criteria and instead encouraged clinicians to use best judgment for virus testing [42]. In some surveillance efforts, travel history was deemed to be less important in risk assessment once community acquisition increased [43].

When planned in 2017, considerations for this capability were primarily concerned with importation of infectious disease, so the scope was focused on travel outside the United States. Presently, the deployed model does extract some location mentions within the continental United States, but the frequency of these extractions and remaining false negatives have not yet been estimated. Such a capability could be useful for aiding containment and tracking of international spread for future epidemics.

Future work would benefit from refining the annotation guideline and training to prevent disagreement like the examples presented here. Furthermore, model training would benefit from additional annotation sampled from other time periods and infectious diseases to assess true validity and improve performance for continued surveillance preparedness. As no data augmentation methods were applied in this study, there are several approaches using keyword or pattern replacement that could improve model performance with available resources [44-46].

Extracting travel history from clinical notes involves the same challenges as any efforts based on secondary use of clinical data. Patient travel history is not always recorded in clinical notes, so this is still an incomplete representation of actual events. Perl and Price [15] emphasized the importance of travel history documentation in the EHR, particularly to enable timely response to emerging pathogens like COVID-19 to protect both patients and frontline health care providers. We note that recording travel history in a structured format would yield more accurate results if faithfully adhered to, but we shall continue to monitor, evaluate, and improve this system as we leverage it to attain a more complete picture of disease for global health. It remains impossible to predict future transition phases in response to COVID-19, but we believe that such a capability could provide value in regions engaged in the phases of prevention or containment.

### Conclusion

We have proposed methods for automated extraction of patient travel history from clinical documents and have demonstrated enhanced capabilities for improving public health systems. This system has been combined with surveillance metrics to inform decision makers on the role of travel history in disease dynamics. It has also been used to reduce a manual chart review to verify whether an infectious disease case may have been imported or locally acquired. These methods have now been deployed to ongoing operations to support enhanced understanding and monitoring. Validity of the system has been sufficient to reduce analyst burden, while computational requirements remain low, allowing thousands of documents to be processed daily. Such capabilities have been leveraged in infectious disease responses, such as Zika and the importation of COVID-19 to the United States, amid dynamically evolving situations. Such systems may aid future efforts to prevent and contain the spread of COVID-19 and other infectious diseases.

## Abbreviations

CDC: Centers for Disease Control and Prevention
CRF: conditional random fields
CUI: concept unique identifiers
EHR: electronic health record
ICD-10: International Classification of Diseases, Tenth Revision
NER: named-entity recognition
NLP: natural language processing
VA: Veterans Affairs
